# Three Successive Infusions: Scientific Insights Into a Traditional Green Tea Drinking Custom

**DOI:** 10.1111/1750-3841.71082

**Published:** 2026-05-06

**Authors:** Xiaoying Zhang, Anas Yusuf, Man Xu, Murtala Bindawa Isah

**Affiliations:** ^1^ Chinese‐German Joint Institute for Natural Product Research, Shaanxi International Cooperation Demonstration Base Shaanxi University of Technology Hanzhong China; ^2^ Centre of Molecular and Environmental Biology (CBMA), Department of Biology University of Minho, Campus de Gualtar Braga Portugal; ^3^ Department of Biomedical Sciences, Ontario Veterinary College University of Guelph Guelph Canada; ^4^ Department of Biochemistry Umaru Musa Yar'adua University Katsina Nigeria

**Keywords:** antioxidant capacity, bioactive compounds, brewing times, green tea, tea drinking customs, tea flavors

## Abstract

This study examines the scientific basis of a traditional multi‐infusion tea‐brewing practice common in Asia by evaluating compositional changes, antioxidant capacity, and sensory attributes across successive green tea infusions. Using UPLC‐MS, GC‐IMS, and elemental profiling, we characterized how repeated extractions alter key tea constituents. Although flavonoids, polyphenols, polysaccharides, and amino acids remained detectable across all infusions, the concentrations of major bioactive compounds including gallic acid, its esters, and L‐theanine declined progressively from the first to the third infusion (GT1–GT3), corresponding with reduced in vivo radical‐scavenging activity. Changes in volatile profiles and sensory evaluation further indicated noticeable shifts in flavor and taste with each infusion. Pearson correlation analysis revealed that most quantified compounds were positively associated with puckery, bitter, and acidic attributes, and negatively associated with sweetness and freshness. Variable Importance in Projection (VIP) analysis identified L‐lysine, L‐tyrosine, epicatechin, myricetin, theophylline, quercetin, and epigallocatechin as key contributors to sensory perception. These findings demonstrate that multiple infusions diversify flavor characteristics while modulating the bioactive profile of green tea, offering insights relevant to product development and consumer brewing practices.

## 1 Introduction

Tea, the infusion product of various parts of the plant *Camellia sinensis*, is the most consumed beverage in the world, second only to water (Venditti et al. 2010). There has been an increase in tea production and tea consumption has grown by 3.5% each year over the past decade, reaching an estimated volume of approximately 6.4 million tons in 2021 (FAO 2022). For over 3000 years, tea has stood as a cultural hallmark in China. Its spread across the globe gave rise to various tea drinking customs and the emergence of well‐recognized terminologies such as Chinese tea culture, Japanese tea culture, British tea culture and others, to capture the uniqueness of tea drinking patterns. In addition, the preferred type of tea and preparation methods also vary across countries. In China, green tea is highly favored, with a traditional preparation method that involves infusing the leaves in hot water for short durations, followed by multiple reinfusions. This practice, known as Gong Fu Cha, is not merely cultural—it modulates the flavor, aroma, and chemical profile of the beverage, and has deep roots in Chinese tea culture and cherished for generations (d'Abbs 2019). Over time, Gong Fu Cha has transcended borders, spreading to neighboring countries like Japan and gaining popularity among the American population (Hendren 2012). On the other hand, in most other countries, including the United Kingdom, Ireland, and Canada, black tea, prepared with boiling water and typically complemented with milk and sugar, is predominantly favored (Venditti et al. 2010; Xiao et al. 2023).

Beyond being a recreational drink, scientific evidence supports the health benefits of drinking tea. Human studies indicate that tea consumption is inversely associated with the risk of developing diabetes mellitus, and its complications (Martin et al. 2017; Meng et al. 2019; Asbaghi et al. 2020). Tea has also demonstrated beneficial effects in managing nonalcoholic liver disease (Tang et al. 2021) and is associated with a decreased risk of breast cancer in women (M. Zhang et al. 2006). The health benefits of tea consumption are attributable to active ingredients belonging to mainly polyphenol, flavonoid and carbohydrate chemical classes (Xiao et al. 2011; Tan et al. 2017; Koláčková et al. 2020). More specifically, (‐)‐epicatechin (EC), (‐)‐epicatechin gallate (ECG), (‐)‐epigallocatechin (EGC), and (‐)‐epigallocatechin gallate (EGCG) (Cabrera et al. 2003; Dai et al. 2017), known for their strong antioxidant properties, have received considerable research attention. These compounds demonstrated the potential to manage human ailments associated with reactive oxygen species (ROS), such as inflammatory diseases and reproductive problems (Roychoudhury et al. 2017). In addition to radical scavenging activities, tea extracts have been shown to activate nuclear factor erythroid 2–related factor 2 (NRF2) signaling and induce the production of antioxidant defenses (Li et al. 2016), relevant for managing cardiovascular diseases, neurodegenerative disorders, and pulmonary diseases (Forman and Zhang 2021). Tea also contains other beneficial compounds including fluoride, caffeine, and essential minerals and nutritious trace minerals (Cabrera et al. 2003). Green tea may be regarded as a functional food and a nutraceutical to be a recommended supplement for lifestyle related diseases (Chacko et al. 2010).

Tea‐drinking culture holds importance at many levels in the tea value chain. Tea contains nonvolatile and volatile compounds derived from precursors including carotenoids, lipids, and glycosides. The compositions and proportions of these compounds confer varying pleasant aromas to tea (Ho et al. 2015). Tea flavor has garnered significant interest from food chemists, with preservation of aroma being a routine measure of tea quality. Various aroma classifications and their characteristic volatile compounds have been reported, including woody, floral, flowery, and fruity aromas attributed to carotenoids; fresh and greenish aromas attributed to lipid derivatives; and sweet, smoky, coffee‐like, green, and chestnut odors attributed to glycosides and products of the Maillard reaction (Ho et al. 2015; Wang et al. 2022). On the other hand, nonvolatile compounds in tea, such as carbohydrates, polyphenols, alkaloids, amino acids, and organic acids, contribute to the sweet and bitter tastes of tea popular among tea drinkers (Wang et al. 2022). Therefore, understanding these diverse chemical compositions is essential for appreciating the multifaceted sensory experience of tea, a quality that tea consumers valued. However, it should be noted that the aroma of this delicate beverage is significantly influenced by the brewing method employed during its preparation.

Brewing, fundamentally, is the process of dissolving soluble components from tea leaves into a solvent, typically water. This process is highly variable, influenced by a range of techniques employed across cultures and for different tea varieties. These different preparation methods significantly impact the taste and phytoconstituents of the final tea product. Previous studies have shown that factors including temperature, time and water/tea material ratio affect the phytoconstituents and sensory properties of green tea (Sharpe et al. 2016; Liu et al. 2018; Cao et al. 2021), Furthermore, key parameters like steeping duration and agitation significantly affect the efficiency and selectivity of compounds extraction (Chong and Nyam 2022; Winiarska‐Mieczan and Baranowska‐Wójcik 2024). For instance, elevated water temperatures generally facilitate the extraction of a broader spectrum of compounds, including both desirable flavor compounds and less desirable bitter components, whereas shorter steeping times may result in a lighter infusion with a higher concentration of volatile aromatics (Chen et al. 2022; Lee and Chambers 2009). Different tea types often require specific brewing protocols to optimize their unique characteristics. Green teas are frequently brewed at lower temperatures to preserve their delicate flavors and minimize the extraction of excessive tannins, which can impart bitterness (Kılıç et al. 2017). Traditional brewing methods, like the Gong Fu Cha and the precise techniques of the Japanese tea ceremony, highlight the cultural importance and sophisticated understanding of how brewing parameters can be manipulated to achieve specific sensory outcomes.

In China, it is culturally known that the different stages of green tea brewing process present different flavors and tastes. Moreover, scientific evidence suggests that the level of mineral and trace elements in tea prepared via this popular preparation method varies across successive infusions (Xiao et al. 2023). However, the effects of multiple infusions on phytoconstituents, organoleptic properties and antioxidant activities of tea infusions has not been explored. Empirical evidence and clinical trials have proven the safety of multiple and sustained tea‐drinking behaviors. Coupled to the aforementioned beneficial phytoconstituents in tea, the multiple brewing method of preparing tea may be a good way to add value to tea products, especially in preventing related diseases among the general populace. The practice of multiple tea infusions is deeply rooted in the culture of Chinese and other East Asian countries, with a focus on enhancing drinking comfort. While it is evident that multiple infusions offer advantages such as resource optimization and optimal tea utilization, the scientific validity and potential health benefits underlying this practice remain unclear. This study aimed to explain whether the traditional “three successive infusions” tea drinking habit has scientific rationality and potential health benefits by investigating the phytochemical, antioxidant, and sensory properties throughout the preparation process. Such understanding is important and meaningful in exploring the value of tea products and promoting rational tea consumption for better health benefits.

## 2 Materials and Methods

### 2.1 Preparation of Tea Infusions and Extracts

**FIGURE 1:**
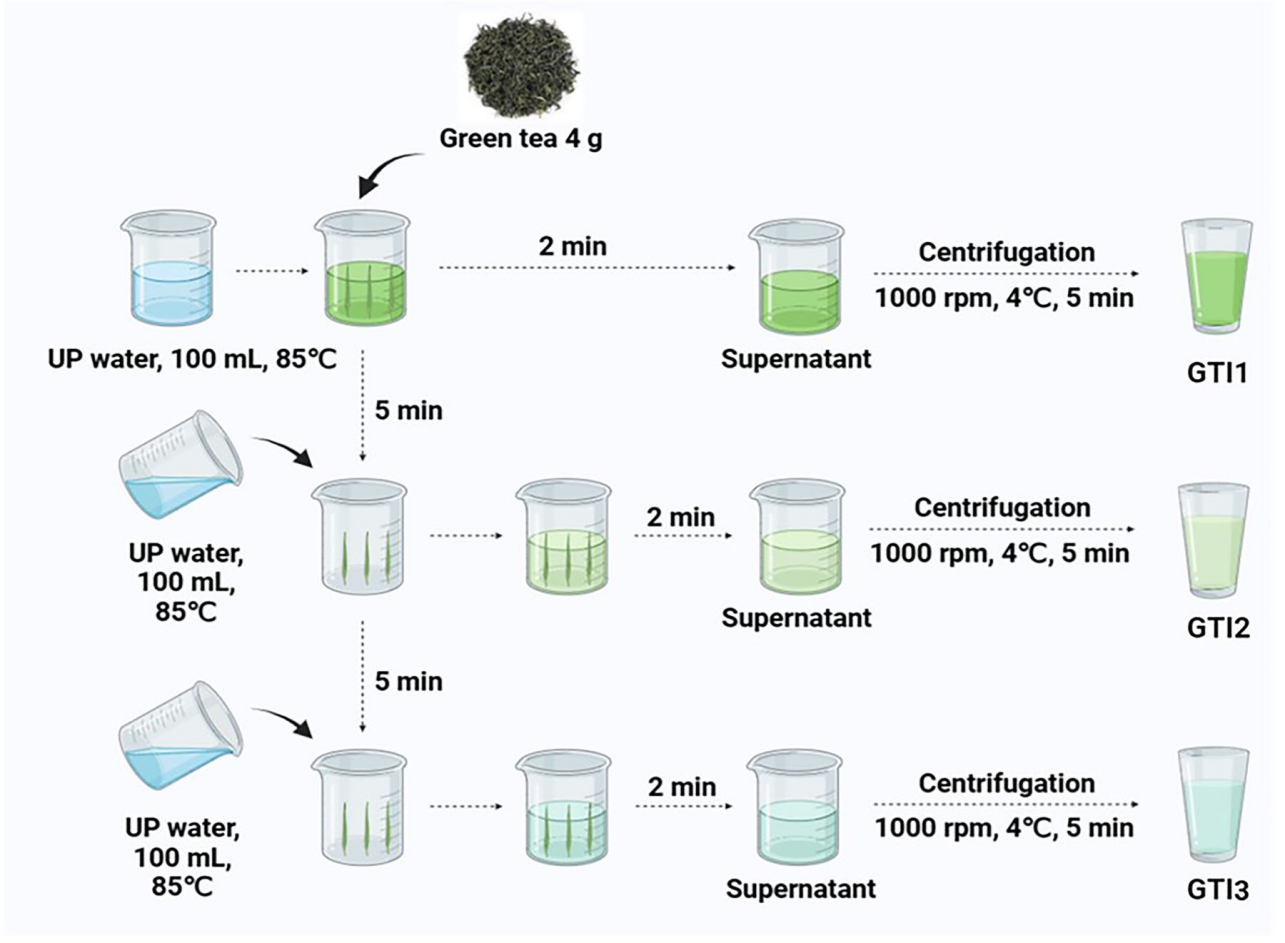
The procedure for the preparation of the tea infusions. This preparation mimics green‐tea making in most parts of China. GTI1, GTI2, and GTI3 represent the teas obtained after the first, second, and third infusions, respectively.

Freshly processed Hanzhong “Xianhao” green tea leaves with particle size of about 180–250 µM were purchased from Xiaoli Tea Shop (Hanzhong, Shaanxi, China). Three successive tea infusions were prepared following the procedure illustrated in Figure 1. Briefly, about 4 g of the tea leaves were infused with pure water (100 mL, 85°C) for 2 min. The infusion was separated from the leaves and clarified by centrifugation (1000 rpm, 4°C, 5 min) to obtain GTI1. The residual leaves were allowed to rest for 5 min before being reinfused with fresh pure water under identical conditions to generate GTI2 and GTI3, each followed by centrifugation. Each tea infusion was prepared from independent batches of tea leaves, with each batch subjected to the same infusion protocol. Unless otherwise stated, three independent replicates (*n* = 3) were prepared for each infusion.

To compare the traditional infusion method with standard laboratory extraction, two additional extracts were prepared: a water extract (WE) and an ethanol extract (EE). For each extract, 1 g of tea was mixed with 25 mL of either double‐distilled water or 70% ethanol and subjected to ultrasonication at 60°C for 40 min (Ultrasonic Cleaner, KunShan Ultrasonic Instruments, Jiangsu, China). To account for batch effects, all tea infusions were prepared in triplicate independently. The mixtures were then centrifuged at 8600 rpm for 5 min at 4°C, after which the supernatants were collected, filtered, and stored at −20°C until further analysis.

### 2.2 Ultra‐High‐Performance Liquid Chromatography‐Mass Spectrometry (UHPLC‐MS/MS) Analysis

Chromatographic separations were achieved using an ultra‐high‐performance liquid chromatograph quadrupole‐high field Orbitrap high‐resolution mass spectrometry (UHPLC‐Q‐Orbitrap‐MS/MS) equipped with a Hypersil GOLD Vanquish C18 column (25002‐052130‐V, 2.1 mm × 150 mm, 1.9 µm, Thermo Scientific, Waltham, MA, USA). Ten microliters of each sample were injected into the liquid chromatograph system. Mobile Phase A was ultrapure water containing 0.1% formic acid. Mobile Phase B was acetonitrile, and the gradient elution program was set as follows: 95%–50% (B) for 0–7 min, 50%–5% (B) for 7–9 min, and 5% (B) held for 9–10 min, at a flow rate of 0.5 mL/min at a column temperature of 35°C. Data acquisition of mass spectrometry was performed in the negative ionization mode, and the spray voltage was 3.1 kV. The flow rates of sheath gas and auxiliary gas were 45 and 10 (in arbitrary units), respectively. The capillary and auxiliary gas heater temperature were 320°C and 300°C, respectively. The S‐lens RF level was 50. Full‐scan MS and data‐dependent MS/MS (ddMS^2^) acquisition were performed with resolutions of 70,000 and 35,000, respectively. The top five most intense precursor ions per full scan were selected for ddMS^2^ with an intensity threshold of 1 × 10^4^. Dynamic exclusion was enabled with an exclusion duration of 10 s. The normalized collision energy was 30%, and the mass scan range was mass‐to‐charge ratio (*m*/*z*) 66.7–1000 (Zeng et al. 2022). For each infusion replicate, analysis was performed with two technical injections, and averaged values were used for statistical analysis. Statistical comparisons were conducted using the replicates as the unit of replication.

Raw data generated from the analysis were converted to the mz/ML format using MS‐Convert (Version: 3.0.24187‐d5e931e). The data were then imported into MZmine (4.2.0) software for mass detection, chromatogram building, deconvolution, noise filtering, peak identification, peak alignment, and peak annotations using an imported spectral library. Further identification and annotation of the compounds were performed using an in‐house database, leveraging average retention time and *m*/*z* values. This process was supplemented with references from the published literatures.

### 2.3 Gas Chromatography‐Ion Mobility Spectroscopy

The gas chromatography‐ion mobility spectroscopy (GC‐IMS) analysis was conducted using a FlavourSpec flavor analyzer (GAS GmbH, Dortmund, Germany) equipped with an MXT‐5 column (15 m × 0.53 mm × 1 µm). The headspace incubation temperature was set at 80°C, with an incubation time of 15 min and an incubation speed of 500 rpm. The injection needle temperature was maintained at 85°C, with an injection volume of 500 µL, and high‐purity N2 was used as the carrier gas in non‐split mode. Throughout the 30‐min analysis, the chromatographic column was held at a constant temperature of 60°C. The carrier gas flow rate started at 2 mL/min for 2 min and linearly increased to 100 mL/min over 18 min, remaining constant for 10 min. For qualitative analysis, the analytical software VOCal was utilized to view the spectra and qualitative data, utilizing the built‐in NIST and IMS databases. The Reporter plug‐in was employed to compare spectral differences between samples, while the Gallery Plot plug‐in generated fingerprints of volatile compounds. In addition, the Dynamic Principal Component Analysis (PCA) plug‐in facilitated cluster analysis and generated PCA spectra of volatile compounds.

### 2.4 Determination of Total Polyphenol Content

The total polyphenols in the samples were determined using the Folin‐phenol colorimetry method (Chu et al. 2002). Gallic acid served as the standard, and a standard curve was constructed using different concentrations of gallic acid (20, 40, 60, 80, and 100 µg/mL). The absorbance was measured at 760 nm using the Epoch 2 Microplate Reader Spectrophotometer. The total polyphenol content in the sample was expressed as milligrams of Gallic Acid Equivalents per gram (mg GAE/g).

### 2.5 Determination of Total Polysaccharide Content

The total polysaccharide content of the samples was determined using the phenol–sulfuric acid method (Fan et al. 2022). Glucose served as the standard, and standard curves were generated using various concentrations of glucose solutions (20, 40, 60, 80, and 100 µg/mL). The absorbance was measured at 490 nm using the Epoch 2 Microplate Reader Spectrophotometer. The total polysaccharide content in the sample was expressed as milligrams of glucose equivalents per gram (mg GE/g).

### 2.6 Determination of Total Flavonoid Content

The total flavonoid content was assessed using sodium nitrite‐aluminum nitrate colorimetry (Xi et al. 2017). Rutin was employed as the standard, and a standard curve was created using various concentrations of rutin (0–250 µg/mL). The absorbance was measured at 510 nm using the Epoch 2 Microplate Reader Spectrophotometer. The total flavonoid content in the sample was expressed as milligrams of rutin equivalents per gram (mg RE/g).

### 2.7 Determination of Free Amino Acids

Amino acid content was determined with reference to the Chinese national standard GB/T 8314‐2013 “Tea‐ Determination of free amino acids content.” Tea samples (1 mL) were pipetted into a 25 mL colorimeter tube, followed by the addition of 0.5 mL of phosphate buffer pH 8.0 and 0.5 mL of a 2% ninhydrin solution. The solutions were heated in a boiling water bath for 15 min. After cooling, the volume of the samples was made up to 25 mL with water and allowed to stand for 10 min. The absorbance of the samples was measured at 570 nm using a 5 mm colorimetric cup with a reagent blank solution as a reference. Three samples of each tea preparation were analyzed in parallel and the results were expressed as (µg/mg dry weight).

### 2.8 Determination of Mineral and Trace Elements

To determine the mineral ion content of the sample, 100 mg of the sample was placed in a nitro boiling tube and digested by open‐vessel nitric acid digestion with 2 mL of nitric acid solution for 1 h at 95°C under atmospheric pressure. Ultrapure water was subsequently added, and the volume was adjusted to 20 mL with dd H_2_O. All samples were prepared in triplicate, and mineral ion content was analyzed using an inductively coupled plasma emission spectrometer (ICP‐OES; PerkinElmer Avio200, MA, USA) to identify the mineral and trace elements present in the samples.

### 2.9 Zebrafish Husbandry, Embryo Collection, and Determination of Antioxidant Activity

Adult wild‐type zebrafish (both male and female) were maintained under standard laboratory conditions. Embryos were obtained through natural spawning, rinsed with embryo medium E3 (5 mM NaCl, 0.17 mM KCl, 0.33 mM CaCl_2_, and 0.33 mM MgSO_4_, pH 7.5), and incubated at 28 ± 1°C. The medium was replaced, and the embryos were washed every 24 h to maintain optimal conditions. All experimental procedures were conducted in compliance with the NIH Guide for the Care and Use of Laboratory Animals (No. 8023, revised in 1996), and approved by the institutional ethical committee, Shaanxi University of Technology (2025‐04).

The antioxidant activity of tea infusions was evaluated based on their effects on paraquat‐induced oxidative stress. Zebrafish larvae at 5 days postfertilization (dpf) were incubated in E3 medium containing either 25 µM paraquat (oxidative stress group) or 25 µM paraquat supplemented with GT1, GT2, or GT3 (treatment groups) (Kim et al. 2022). A control group was maintained in embryo medium alone. The medium was refreshed, and larvae were washed every 24 h. At 8 dpf, ROS levels in the larvae were assessed using 10 µM 2′,7′‐dichlorofluorescein diacetate (DCFH_2_‐DA). The larvae were incubated with the dye for 1 h at 28 ± 1°C in the dark, followed by three washes with E3 medium to remove excess dye (Kim et al. 2022). Fluorescence images of all embryos were captured using an inverted phase‐contrast microscope (OLYMPUS CKX53, Tokyo, Japan). The pixel intensity of the fluorescence images, indicative of ROS levels, was quantified using ImageJ Pro Plus software (Rawak Software Inc., Stuttgart, Germany). Each treatment group consisted of four independent wells, with 15 larvae per well. Larvae within each well were considered subsamples, and statistical analyses were performed using wells as the unit of replication.

### 2.10 Electronic Tongue Analysis

The tea infusions were evaluated using an electronic tongue (SA402B, Insent Intelligent Sensor Technology, Inc., Japan). The electronic tongue was sampled every 1 s, with a 5‐s sample preparation time, 150 s of continuous sampling, 30 s allocated for cleaning, 10 s for return to 0, and internal and injection flows set at 260 mL/min. All analysis were conducted in technical triplicates for each infusion replicate.

### 2.11 Statistical Analysis

All data were presented as mean ± SD. Variations among different groups were compared using analysis of variance (ANOVA) with a significance threshold of *p < 0.05, **p < 0.01, and ***p < 0.001, conducted using GraphPad Prism software version 8 (GraphPad Software, Boston, USA), followed by Tukey's post hoc test. Partial least squares regression (PLSR) analysis was used to analyze the dynamic changes of sensory characteristics of the tea infusions with changes in chemical components using SIMCA 14.1.

## 3 Results

### 3.1 UHPLC Analysis and Quantification of TPC, TFC, TPS, and FAA Revealed Compositional Variations Across Successive Infusions

**FIGURE 2:**
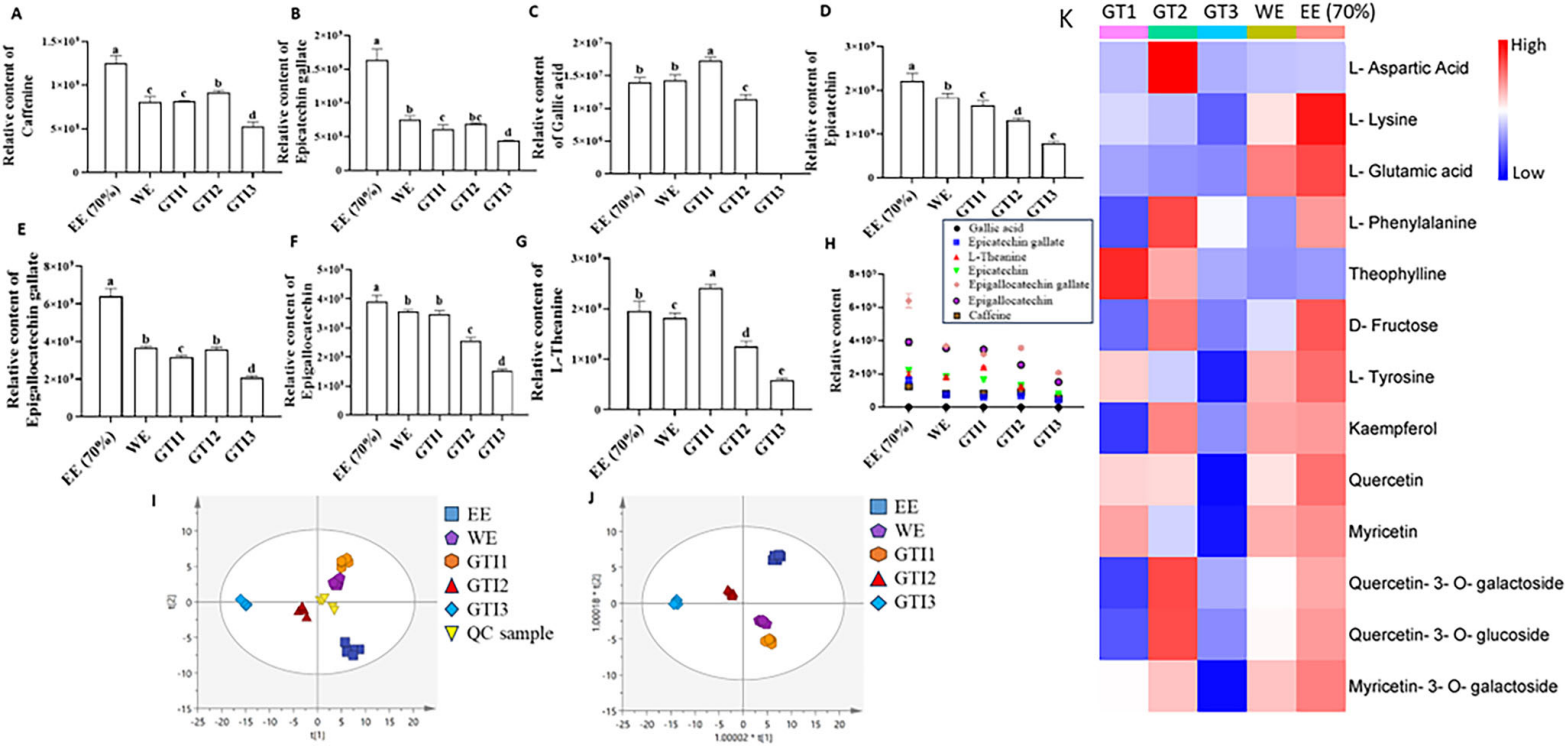
Changes in relative content of key bioactive compound levels across successive infusions of green tea. (A–G) show the relative contents of major bioactive compounds across different green tea infusions and extracts: ethanolic extract (70%) (EE), water extract (WE), and three successive green tea infusions (GTI1, GTI2, and GTI3). The compounds analyzed include: (A) caffeine, (B) epicatechin gallate, (C) gallic acid, (D) epicatechin, (E) epigallocatechin gallate, (F) epigallocatechin, and (G) L‐theanine. The data presented as mean ± SD, with different letters above bars indicating statistically significant differences (*p* < 0.05) among the groups. (H) Demonstrate changes in relative content for each bioactive compound across the successive extractions. (I) and (J) are the principal component analysis (PCA) and partial least squares discriminant (PLS‐DA) plots that illustrate the clustering of samples based on compound composition, with clear separation of between the groups, indicating distinct biochemical profiles among EE, WE, and GTI samples. Quality control (QC) samples demonstrate the consistency of the UHPLC analytical process. (K) Heatmap showing normalized (auto‐scaled) intensity values of other compounds detected across the tea infusions.

Relative abundance was used to assess the major compounds in green tea (Figure 2). Caffeine content was significantly higher in GTI2 than in other infusions, with GTI3 showing the lowest level. The relative caffeine levels in the WE and GTI1 were similar, with no significant difference (*p *> 0.05) observed between the two infusions, while the ethanolic extract had the highest caffeine level (Figure 2A). Although there was no significant difference in the relative amount of epicatechin gallate between GTI1 and GTI2, GTI2 had a slightly higher level, while GTI3 showed the lowest content. The ethanolic extract contained the highest level of epicatechin gallate (Figure 2B). Gallic acid content was significantly higher in GTI1 than in all other infusions and was undetectable in GTI3, suggesting it was either absent or below the detection limit (Figure 2C).

EC showed a decreasing trend across the infusions (Figure 2D). Epigallocatechin gallate was significantly higher in GTI2 than in the WE, GTI1, and GTI3, while the ethanolic extract had relatively higher levels than all infusions (Figure 2E). The relative composition of EGC was significantly higher in GTI1 (*p *< 0.05) compared to GTI2 and GTI3, though similar to the WE (*p *> 0.05); the ethanolic extract showed a significantly higher content than all infusions (*p *< 0.05) (Figure 2F). GTI1 exhibited a significantly higher amount of L‐theanine than the ethanolic and WEs, GTI2, and GTI3 (Figure 2G). Overall, the major green tea compounds demonstrated distinct patterns across the infusions and comparative extracts (Figure 2H).

Principal component analysis (PCA) and partial least squares discriminant analysis (PLS‐DA) of the major tea bioactive components (gallic acid, EC, epicatechin gallate, EGC, epigallocatechin gallate, L‐theanine, and caffeine) revealed compositional variations among the different preparations. The WE and GTI1 showed similar compositions of the analyzed compounds (Figure 2I,J, respectively), with the exception of L‐theanine and gallic acid in GTI1. Heatmap was used to visualize the distribution and changes of other compounds across the successive tea infusions (Figure 2K). GT2 exhibited high abundance of L‐aspartic acid, and L‐phenylalanine, consistent with the result of the analysis of free amino acid (FAA) content (Figure S1D). Furthermore, GTI1 had the highest total polyphenol content among the three teas, although not significantly different from the water and EEs (Figure S1A). GTI2 and GTI3 contained 73% and 42% of GTI1's total polyphenol content, respectively. Total polysaccharide and flavonoid contents followed a similar trend, with significant differences among all samples, ordered as: EE > WE > GTI1> GTI2> GTI3 (Figure S1B,C). GTI2 had the highest total FAA content, closely followed by GTI1 (Figure S1D). A single hot water infusion was as effective as, or even more effective than, ultrasonic water extraction in extracting these phytochemicals, particularly FAAs.

### 3.2 Antioxidant Effects of Tea Infusions on Paraquat‐Induced Oxidative Stress in Zebrafish Larvae

**FIGURE 3:**
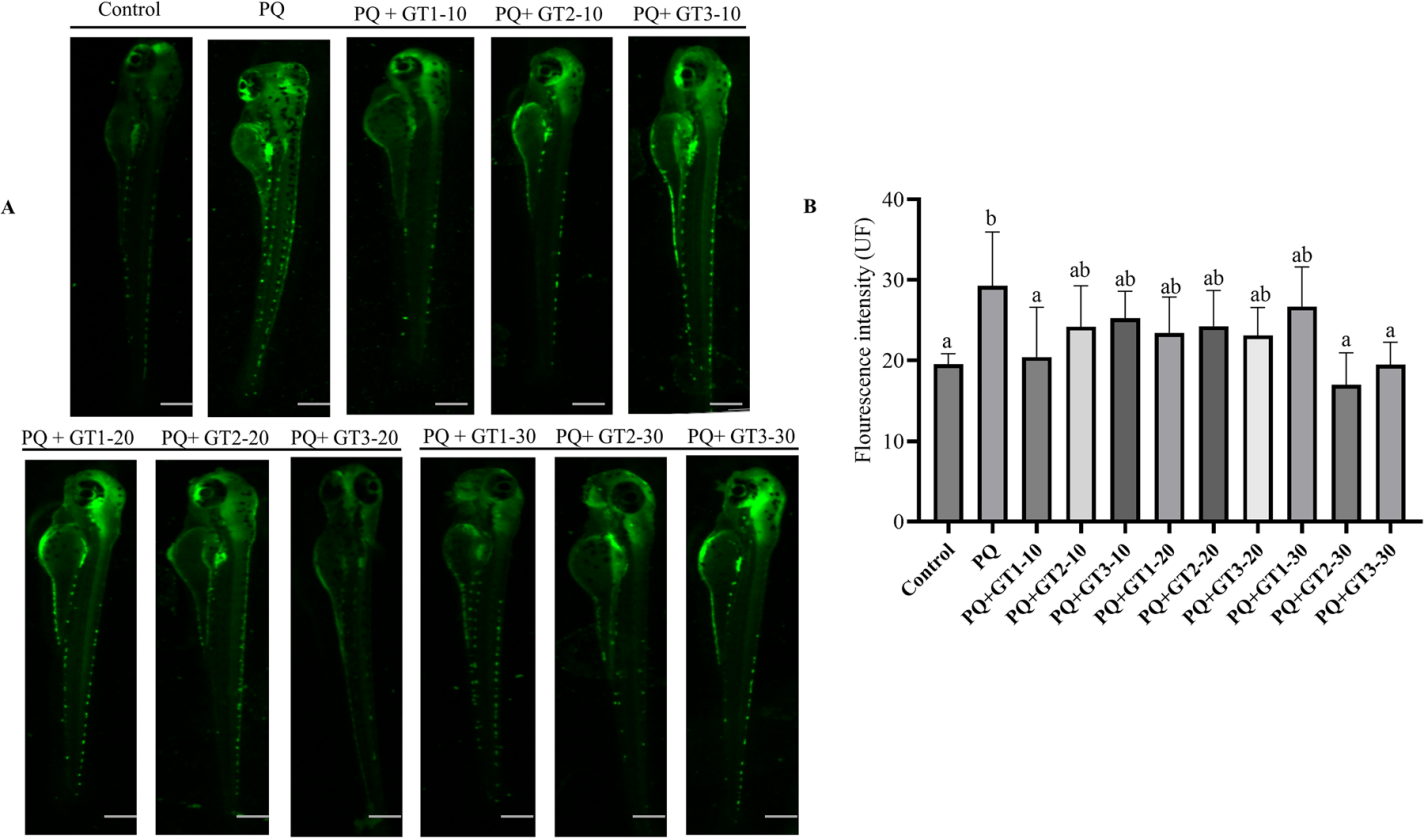
Antioxidant effects of tea infusions on paraquat‐induced oxidative stress in zebrafish larvae. (A) Representative images of zebrafish larvae at 8 days postfertilization treated with paraquat (25 µM) alone or in combination with varying amounts (10, 20, and 30 µL/mL) of GT1, GT2, or GT3 tea infusions. (B) Quantitative analysis of ROS level in zebrafish larvae. Bars with different letters indicate statistically significant differences between groups (*p* < 0.05). Values are expressed as mean ± standard deviation (*n* = 3).

To evaluate the antioxidant activity of different tea infusions, zebrafish larvae were exposed to paraquat (25 µM) to induce oxidative stress, followed by treatment with varying amounts (10, 20, and 30 µL) of each tea infusion. A significant increase in fluorescence intensity was observed in the paraquat treated group compared with control and tea treated groups (*p *< 0.05), indicating elevated level of ROS (Figure 3A). In contrast, treatment with all three tea infusions resulted in a dose‐dependent reduction in ROS levels (Figure 3B). Treatment with 10 µL of GT1 in a total volume of 1 mL E3 medium resulted in a significant reduction in the level of ROS compared to the paraquat‐treated group (*p *< 0.05). increasing the tea infusion to 20 or 30 µL reduced ROS compared to the paraquat‐treated group, but the reduction was less pronounced than with 10 µL and not statistically significant (*p *> 0.05). These findings suggest the presence of some constituents in GT1 that may exert pro‐oxidant effects, influencing the overall antioxidant activity of the tea and potentially leading to the loss of ROS scavenging efficiency at higher concentrations. On the other hand, GT2 and GT3 showed a significant reduction in ROS levels only when the amount was increased to 30 µL, further highlighting the dose‐dependent effects of the tea. This observation is consistent with the reduction in phytochemical constituents across the successive tea infusions.

### 3.3 Teas From Successive Infusions Displayed Varying Aromas and Flavors

**FIGURE 4:**
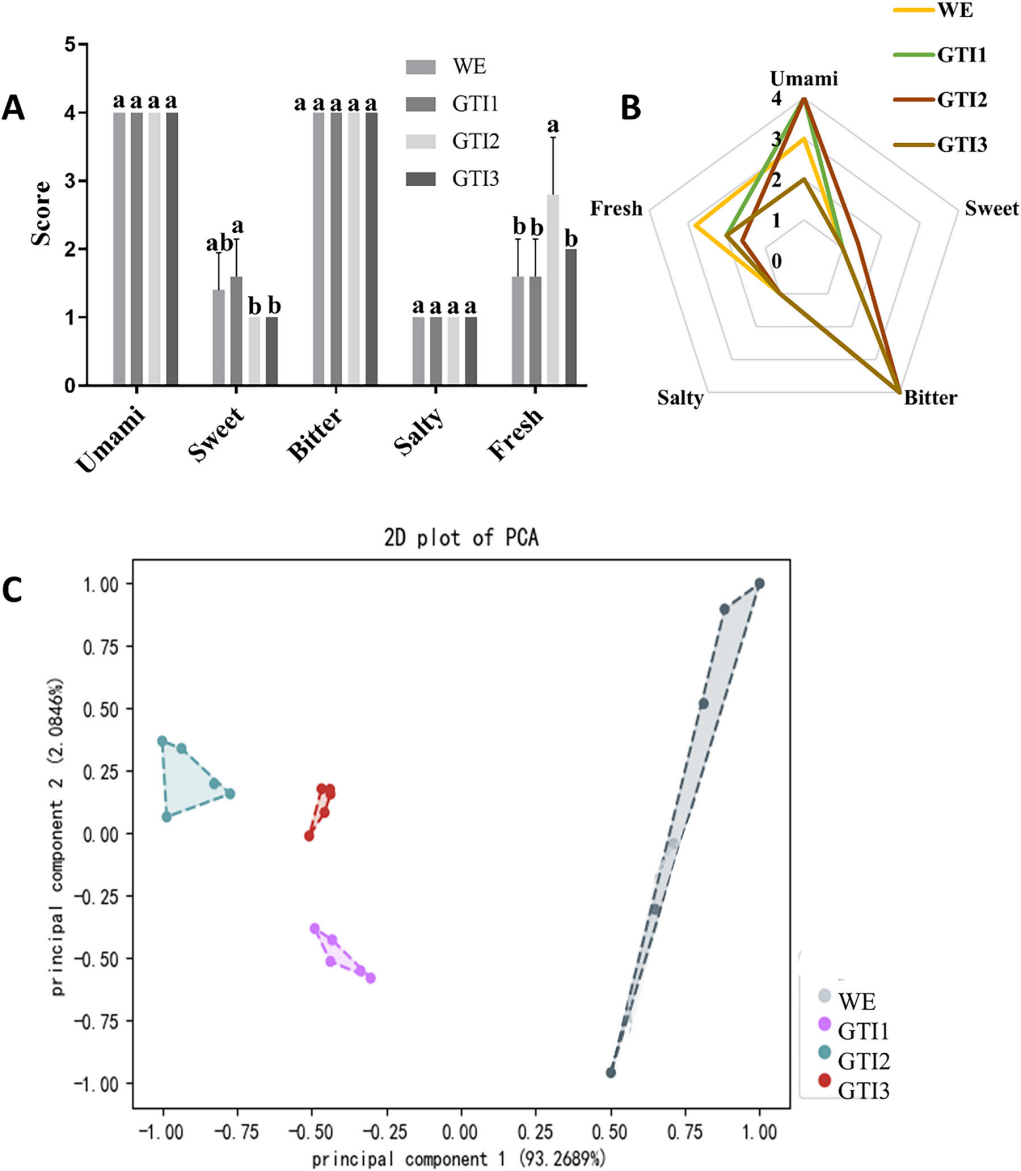
Sensory evaluation of the tea infusions using electronic tongue. Analysis of five flavor attributes using an electronic tongue (A and B) and a 2D PCA plot demonstrating the separation of tea infusions based on sensory attributes (C). Values are expressed as mean ± SD (*n* = 5 for the electronic tongue).

The sensory profile of green tea across successive infusions revealed notable differences in flavor and aroma characteristics as assessed through both electronic tongue. The electronic tongue evaluation indicated significant differences in the five primary taste dimensions of umami, sweetness, bitterness, saltiness, and freshness (Figure 4A). Among these, umami and bitterness scored the highest across all infusions, with GTI1 displaying a significantly higher sweetness score (*p *< 0.05) compared to other infusions. The radar plot (Figure 4B) illustrated the sensory attributes including umami, sweet, bitter, salty, and freshness of the three successive tea infusions and the WE. A distinct variation in the intensity of these attributes was observed across the samples, highlighting the progressive changes in sensory characteristics during successive infusions. GT1 exhibited the highest scores for umami, sweet, and fresh attributes, reflecting a rich flavor profile in the first infusion. These attributes gradually diminished in GT2, consistent with the depletion of key flavor‐contributing compounds across successive infusions. Conversely, bitterness increased slightly in GT2 likely driven by the observed rise in caffeine, EGCG, and ECG (P. Yu et al. 2014). These findings suggest that the first infusion captures the most taste substances, while successive infusions show a shift in flavor dynamics due to the reduction in polyphenols and the relative dominance of amino acids. The sensory variations among the infusions aligned with changes in volatile compound profiles, as observed in the chemical analysis. PCA (Figure 4C) further supports these findings, showing clear clustering patterns for WE, GT1, GT2, and GT3, reflecting distinct sensory and compositional differences. GT1 exhibited partial overlap with WE, indicating shared flavor profiles, while GT2 and GT3 showed significant divergence, representing the depletion of flavor‐related compounds across successive infusions. The first two principal components explained 93.27% and 2.87% of the variance, respectively, confirming the distinctiveness of each infusion's flavor profile. Overall, successive infusions influenced the sensory attributes of green tea, with GTI1 characterized by stronger sweetness and freshness.

### 3.4 Fingerprints Analysis of Volatile Compounds Across the Tea Infusions

**FIGURE 5:**
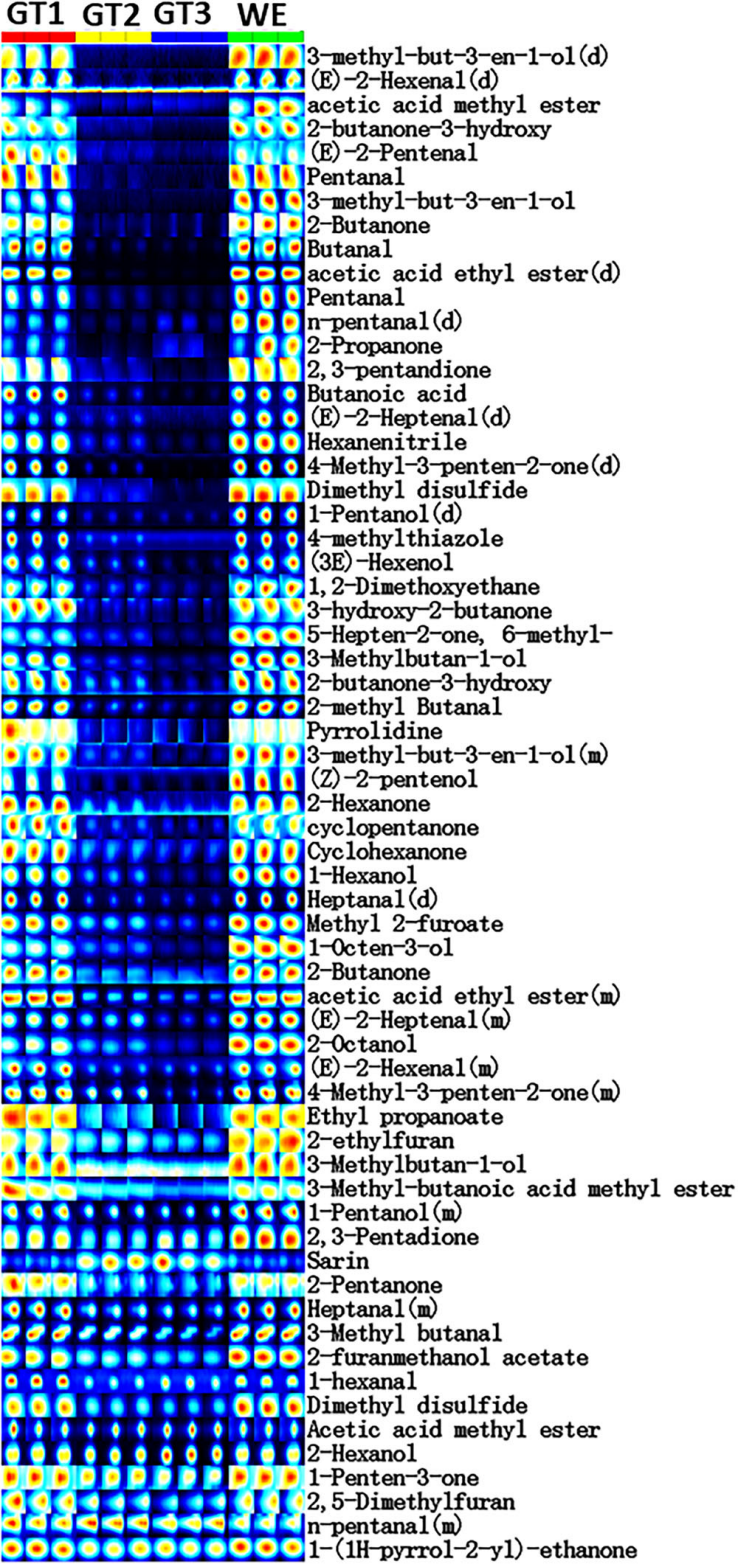
Fingerprints of volatile compounds across the tea infusions. Gallery plot showing the distribution and relative intensities of volatile compounds in three successive tea infusions (GT1, GT2, and GT3) and the water extract (WE). Each row corresponds to a specific volatile compound, while the columns represent different samples. The color intensity reflects the abundance of each compound, with warmer color (yellow to red) indicating higher abundance and cooler colors (blue) representing lower abundance. Where (d) and (m) signify dimer and monomer, respectively.

**FIGURE 6:**
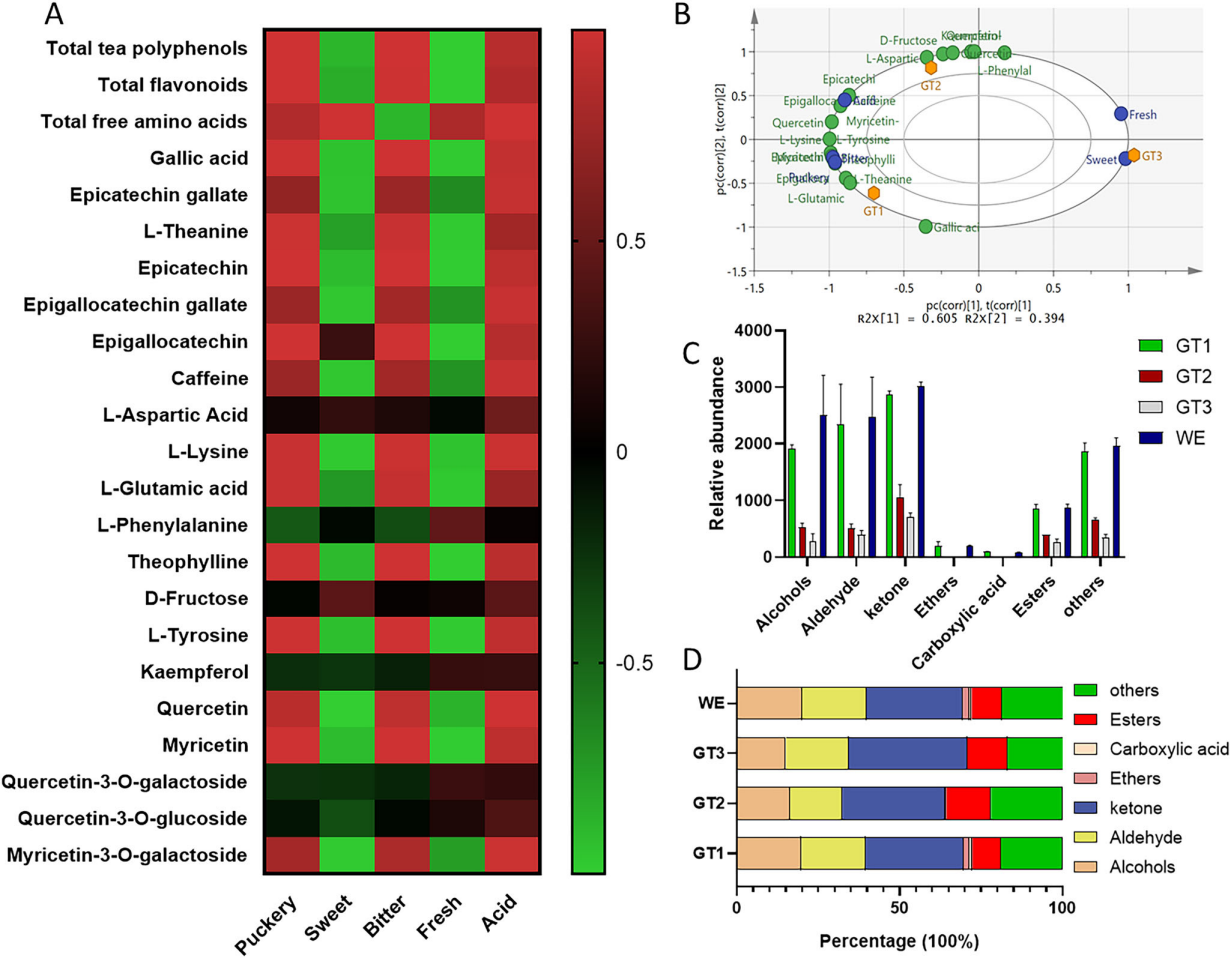
Correlation‐based sensory and relative abundance of volatile compounds across the three infusions. (A) Pearson correlations between key flavor compounds and five sensory attributes (puckery, sweet, bitter, fresh, and acid) across successive tea infusions (GT1, GT2, and GT3). (B) PLSR biplot illustrating dynamic correlations between sensory attributes and chemical compounds across sequential tea infusions (GT1–GT3), highlighting the progressive shifts from bitter‐astringent compounds toward sweet‐fresh. (C) Relative abundance of volatile compounds across the tea infusions (GT1, GT2, and GT3). (D) Percentage distribution of volatile compound classes across the tea infusions.

To observe the changes in the relative abundance of volatile compounds across successive tea infusions, a gallery plot was utilized (Figure 5). The signal intensity of each compound across three successive infusions is represented by spots in the same row, while spots in the same column indicate the relative abundance of compounds within the same sample. Brighter colors correspond to stronger signal peaks and higher relative content. Variations in volatile compounds were further analyzed using two‐dimensional (2D) spectra and three‐dimensional (3D) topographic plots (Figure S2A,B). In the 3D plot, the *x*, *y*, and *z*‐axes represent drift time, retention time, and ionic signal intensity, respectively, with each point corresponding to a volatile compound, including monomers or dimers. The analyses highlight significant variations in signal intensities and volatile compound content across successive tea infusions. The identified compounds belong to classes such as alcohols, aldehydes, alkenes, ketones, ethers, esters, and other volatile classes (Table S2). The relative abundance of most compounds was higher in GT1, except for 1‐(1H‐pyrrol‐2‐yl)‐ethanone, sarin, furan‐2‐ylmethyl acetate, pentanal, and 2‐hexanol, which showed higher relative abundance in GT2 and GT3. Ketones displayed the highest relative abundance and proportion, followed by aldehydes and then alcohols, with a decreasing trend from GT1 to GT3 (Figure 6C,D). These differences in the relative abundance of volatile compounds across successive infusions contribute to the observed variations in the aroma and sensory profiles of the tea infusions. The WE and GT1 displayed similar volatile compound profiles, highlighting the compositional overlap between these two samples

The volatile compound profiles of WE and GT1 were notably similar, indicating compositional overlap between these two samples. The PCA plot (Figure S3) visualizes the distribution of volatile compounds across three successive tea infusions and the WE. Principal Component 1 (PC1) and Principal Component 4 (PC4) explain 90% and 1% of the variance, respectively, capturing both major and subtle data variability. The clear separation among successive infusions in the PCA plot highlights significant differences in volatile compound profiles. PC1 primarily reflects the progressive depletion of volatile compounds from GT1 to GT3, while PC4, accounting for minor variance, likely represents subtle differences in specific compounds, as evidenced by the distinct clustering of the WE. The unique clustering of WE confirm its different volatile composition compared to the infusions. These results highlight the dynamic changes in chemical composition across infusions, which influence the tea's aroma sensory attributes.

### 3.5 Analysis of Key Flavor Compounds and Dynamic Changes in Sensory Attributes Across the Tea Infusions

Pearson correlation were employed to evaluate the general associations between key flavor compounds and each sensory attribute across successive tea infusions (Figure 6A). The result revealed that the majority of compounds exhibited positive correlations with puckery, bitter, and acidic taste attributes, while demonstrating negative correlations with sweetness and freshness. PLSR analysis effectively captured the progressive sensory transformations occurring over sequential tea infusions. The sensory attributes displayed distinct, infusion‐dependent trends. The two‐component PLSR model explained variations in both sensory attributes and compound concentrations. The first two latent variables accounted for 60.5% (PC1) and 39.4% (PC2) of the variation in the compounds data, confirming effective dimensionality reduction while retaining essential compounds information. Furthermore, the separation of infusions along PC1 demonstrated systematic and statistically significant differences in sensory profiles related directly to changes in chemical compositions (Figure S4A,B). Hotelling's T^2^ ellipse confirmed no statistical outliers, highlighting the model reliability. The Variable Importance in Projection (VIP) scores further validated model stability, highlighting specific compounds significantly influencing sensory perceptions (VIP ≥ 1), including L‐lysine, L‐tyrosine, EC, myricetin, theophylline, quercetin, and EGC (Figure S4C). PLSR biplot analysis (Figure 6B) further provided an integrative visualization of how sensory attributes dynamically correlated with specific chemical compounds across the three tea infusions. GT1 was distinctly characterized by its pronounced bitterness and puckery, correlating strongly with elevated levels of catechins (epigallocatechin gallate, epicatechin gallate), gallic acid, caffeine, and certain amino acids (L‐theanine, L‐glutamic acid, and L‐lysine). GT2 occupied an intermediate position, reflecting transitional chemical profiles as certain compounds were moderately depleted resulting in a pronounce effect of other compounds. In contrast, GT3 was closely associated with enhanced sweetness and freshness, attributable to higher relative concentrations of kaempferol, quercetin, D‐fructose, L‐phenylalanine, and L‐aspartic acid. This clear clustering highlights systematic chemical and sensory transitions among infusions.

## 4 Discussion

The sensory differentiation observed across the tea infusions (GT1–GT3) arises from a synergistic interaction between nonvolatile and volatile metabolites, as revealed by the integrated analysis of e‐tongue measurements, GC‐IMS profiling, and multivariate statistics. The pronounced bitter and puckery profile characteristic of GT1–GT2 could be primarily associated with the dominance of nonvolatile polyphenolic compounds, including total tea polyphenols, EC, and EGC, which showed strong positive correlations with bitterness and astringency in the correlation heatmap (Figure 6A). In contrast, the progressive shift toward the fresher and sweeter sensory profile observed in GT3 coincides with a reduced relative contribution of polyphenols and an increased influence of amino acids and volatile aroma compounds. GC‐IMS analysis further indicate that volatile ketones and related aroma‐active compounds could be associated with freshness perception in the GT3, while VIP analysis (VIP > 1.0) identifies L‐lysine, L‐tyrosine, and myricetin as key discriminators driving sensory variance among infusions. Together, these results demonstrate that the transition from the bitter and puckery profile of GT1 through GT2 to the fresher and sweeter profile of GT3 is driven not by a single compound class, but by a coordinated shift in nonvolatile composition and aroma‐active volatiles that collectively shape taste perception.

While green tea is well‐recognized for its abundance of phytoconstituents, the efficiency of tea preparation methods significantly influences the extraction of beneficial compounds (Xu et al. 2018). In this study, multiple infusions yielded higher total polyphenol content compared to ethanol and water extractions performed under laboratory conditions. This finding aligns with the study by D.‐J. Yang et al. (2007), who demonstrated that multiple infusions extract greater quantities of bioactive compounds compared to a single brewing procedure corresponding to the total time of multiple infusions, consistent with our initial findings (data not shown). This phenomenon may be explained by mass transfer kinetics and concentration gradient–driven diffusion. During a single long‐duration steeping, the extraction process rapidly approaches chemical equilibrium, at which point the concentration gradient between the tea leaf matrix and the surrounding solvent diminishes, limiting further solute diffusion (Khan et al. 2025). In contrast to successive infusions, where fresh solvent is introduced at each brewing step, thereby reestablishing a steep concentration gradient that promotes continued diffusion of secondary metabolites from the leaf matrix. As a result, compounds that may remain partially retained within the leaves under steady‐state conditions can be progressively extracted across multiple infusions. Our findings here provide a mechanistic basis for traditional multi‐infusion practices, and supporting their effectiveness in maximizing the recovery of tea phytoconstituents under real‐world consumption condition.

The multiple infusion method employed here efficiently extracted major phytoconstituents of tea, including GA, L‐theanine, EC, EGC, ECG, and EGCG, along with polyphenols, polysaccharides, flavonoids, and amino acids, aligning with findings from previous studies (Pérez‐Burillo 2018; Liu et al. 2018; Cao et al. 2021). However, the current results diverge from those of Horžić et al. (2009), who reported sequential 50% reductions in caffeine content with each successive infusion at temperatures ranging from 80°C–100°C and a 3‐min infusion duration. In contrast, this study observed an increase in caffeine content during the second infusion (GT2), which may be attributed to the shorter 2‐min infusion time employed here. This observation aligns with the mechanistic findings of Sharif et al. (2013), who demonstrated a positive correlation between caffeine extraction and prolonged infusion times, suggesting that the increase in GT2 could reflect an extended effective extraction period, given that the same temperature and tea leaves used in the first infusion (GT1) were maintained. Interestingly, D.‐J. Yang et al. (2007) reported an increase in caffeine content in the second infusion at 70°C, whereas raising the temperature to 85°C resulted in higher caffeine yields in the first infusion compared to subsequent infusions. It should be noted that the extraction of caffeine and other compounds in tea infusions is influenced by additional factors, including surface area and tea leaf‐to‐water ratio, as highlighted by Deka et al. 2020. In addition, previous studies have shown that green tea yields approximately 24 mg, 29, and 39 of caffeine in 230 mL of tea brewed for 1, 3, and 5 min, respectively (Chin et al. 2008).

Nawrot et al. (2003) provided evidence‐based recommendations for daily caffeine intake across different population groups. For healthy adults, a moderate daily caffeine consumption of up to 400 mg/day (equivalent to approximately 6 mg/kg body weight/day for a 65‐kg individual) is considered safe. The recommended daily caffeine intake in women is slightly lower, at ≤ 300 mg/day (equivalent to approximately 4.6 mg/kg body weight/day for a 65‐kg individual). Given the positive correlation between caffeine content and brewing time, consuming green tea prepared using this traditional may contribute significantly to daily intake, especially with heavy consumption. This raises potential health concerns, as excessive caffeine consumption may have detrimental effects on health. Caffeine may impair glucose tolerance (Johnston et al. 2003) and extended caffeine consumption has been associated with migraine (L. Zhang et al. 2023). Despite the lower caffeine content in green tea relative to other widely consumed beverages such as black tea and coffee, the benefit of consuming green tea must also be advocated with due consideration of the level of other ingredients present. In addition, some mineral and trace elements may accumulate in teas above their safe levels for human consumption, as reported in a recent study (Xiao et al. 2023). However, in this study, the levels of all mineral and trace elements investigated are found to be within their safe limits as identified by the Chinese National Standard GB 2762–2017 (Table S1).

Compared to other types of tea (white, yellow, oolong, black, and dark tea), green tea stands out for possessing the highest antioxidant activity due to its elevated levels of relevant phytochemicals (Pastoriza et al. 2017). Active ingredients like polyphenols and flavonoids, particularly EGCG, exhibit potent antioxidant properties, which play a critical role in mitigating inflammatory diseases and oxidative stress‐related conditions (Cabrera et al. 2003; Xiao et al. 2011). In a previous study, the antioxidant properties of green tea showed an increasing trend as the brewing temperature and time increased, indicating the heat stability of the responsible phytochemicals (Liu et al. 2018). The antioxidant properties of the successive tea infusions showed a declining trend, similar to previous studies (Han et al. 2018; J. Yu and Li 2021), and this could be explained by the decreasing trend of the major components of tea with high antioxidant properties, namely: ECG, EGCG, GA, and EC, as well as other polyphenols and flavonoids (Figure 3). A volume of 10 µL of the first infusion effectively reduced oxidative stress induced by paraquat in treated animals. In contrast, comparable effects were only achieved with the second and third infusions when the volume was increased to 30 µL, indicating a reduction in the total antioxidant compounds. Despite this decline, it is noteworthy that significant levels of these essential phytochemicals persist even in the third infusion, suggesting that multiple infusions of green tea continue to provide antioxidative benefits. It should be emphasized that the antioxidant assays were conducted using fixed infusion volumes, a design intended to reflect real‐world tea consumption patterns in which intake is determined by volume rather than standardized phytochemical doses. While this approach enhances translational relevance, it limits direct pharmacological comparisons based on equimolar concentrations of individual compounds among GT1, GT2, and GT3. Accordingly, the observed differences in ROS modulation likely arise from variations in overall infusion composition and effective phytochemical exposure. Future studies aimed at assessing relative efficacy or dose–response relationships may therefore benefit from normalizing treatment concentrations using total polyphenol content or representative marker compounds like EGCG.

The amino acid content in tea infusion has been shown to depend on several factors, including temperature, extraction time, the water‐to‐tea ratio, and tea particle size (Vuong et al. 2011). Notably, around 50% of the total amino acid content in the tea infusion can only be extracted after the first 2 min of infusion (Horanni and Engelhardt 2013). These findings align with the results of the current study, where the maximum amino acid content was observed after the first infusion. This phenomenon can be attributed to several factors, particularly the solubility of amino acids. In GT1, the L‐theanine content was higher than in GT2 and GT3, likely due to its high solubility. In contrast, other amino acids may remain trapped within the leaf matrix and are not fully released. The higher amino acid content observed in GT2 can be explained by the structural relaxation of the tea leaves during prolonged soaking and exposure to hot water, which facilitates the further diffusion of amino acids. Moreover, protein hydrolysis in tea, which releases FAAs, may be hindered by interactions between proteins and polyphenols. Previous research suggests that optimal hydrolysis occurs only when the polyphenol concentration is below 5 mg/mL (Xu et al. 2013). This finding is consistent with the current study, where the polyphenol concentration in GT2 was lower compared to GT1, potentially enabling a greater release of amino acids.

Although there are significant taste interactions among constituents of green tea to produce the bitterness, astringency, and sweet aftertaste (P. Yu et al. 2014; Y. Zhang 2016), catechins, have been found to be the major contributors to the puckery and bitterness (Narukawa et al. 2011). The bitterness and puckery of GTI1 reflect the higher content of catechins compared to the other infusions (Scharbert and Hofmann 2005). Furthermore, amino acids contribute to the fresh and sweet taste of tea, playing a key role in enhancing the overall refreshing quality of tea and mitigating its bitterness. Most of the identified compounds positively correlated with puckery, bitter, and acid, while exhibiting negative correlations with sweetness and freshness, with notable exceptions including L‐aspartic acid, D‐fructose, and L‐phenylalanine, which showed positive correlations with sweetness and freshness. The absence or lack of perception of sweetness and freshness in GT1 and relatively in GT2 indicate suppression of these attributes, likely due to the dominance of compounds caffeine and catechins (Scharbert and Hofmann 2005), which negatively correlate with these attributes. Furthermore, the observed significant improvement in sweetness and freshness scores from GT1 to GT3, suggests a reduction in suppression, potentially driven by decreasing concentrations of negatively correlated compounds. Overall, the key substances driving sensory quality differences across the infusions include L‐lysine, L‐tyrosine, EC, myricetin, theophylline quercetin, and EGC.

Tea nonvolatile and volatile compounds are derived from carotenoids, lipids, and glycosides, contributing to its diverse aroma ranging from woody to fruity, while nonvolatile compounds, including carbohydrates and polyphenols, offer sweet and bitter tastes (Wang et al. 2022). These compounds' composition varies based on factors such as cultivation, processing, storage, and preparation methods (Fang et al. 2024) and their interaction influences tea aroma perception. Thus, as the volatile constituents of tea are dynamically changed with successive infusions, varying flavors were obtained, yielding favorable and unique aromas for the tea obtained at each infusion stage.

Tea is widely recognized for its health benefits, primarily attributed to its polysaccharide and polyphenol content. Tea polysaccharides have demonstrated significant antidiabetic properties (Xiao et al. 2011), while tea extracts have been shown to activate NRF2 signaling, a pathway critical in combating cardiovascular and neurodegenerative disorders (Li et al. 2016; Forman and Zhang 2021). In addition, tea contains fluoride, caffeine, and essential minerals, further establishing its role as a functional food and a valuable supplement for managing lifestyle‐related diseases (Cabrera et al. 2003; Chacko et al. 2010). This study highlights the advantages of multiple infusions of tea, demonstrating a notable benefit: the cumulative extraction of polyphenols, flavonoids, and polysaccharides surpasses the total content obtained through conventional laboratory extraction methods. This shows the potential of traditional tea preparation methods to maximize the health‐promoting properties of tea.

## 5 Conclusion

This study provides a mechanistic and sensory‐based evaluation of the traditional multiple successive infusion method for green tea preparation, demonstrating that repeated infusions do not merely dilute tea constituents but instead drive a dynamic redistribution of bioactive and aroma‐active compounds. Through the integrated application of electronic tongue analysis, GC‐IMS profiling, and multivariate statistical modeling, we show that successive infusions induce a systematic transition from a bitter and puckery sensory profile dominated by polyphenols, to a more balanced chemical extraction in the GT2, and ultimately toward a fresher and sweeter sensory perception in the GT3, characterized by a relatively greater contribution of amino acids and volatile compounds.

Notably, GT2 exhibited the highest overall extraction efficiency for key bioactive constituents, including caffeine, EGCG, ECG, and total amino acids, highlighting the second infusion as a critical stage for maximizing chemical yield. In contrast, GT3 with lower total polyphenol levels and higher ketone composition, demonstrated enhanced sensory freshness and sweetness, highlighting the importance of volatile and nonvolatile interactions in shaping tea flavor beyond absolute compound concentrations. These findings challenge the conventional emphasis on single‐infusion brewing by revealing that optimal sensory quality and bioactive delivery emerge from the collective effects of successive infusions rather than from a single preparation step.

Some limitations should be acknowledged. First, this study was conducted using a single green tea cultivar under standardized water conditions; therefore, variations in water mineral composition, pH, and leaf processing methods like steaming or pan‐firing may significantly influence extraction kinetics and sensory outcomes. In addition, the cumulative caffeine intake associated with multiple infusions warrants further investigation to inform evidence‐based guidelines for functional tea consumption and safety evaluation.

Overall, this work bridges traditional tea‐brewing practices with modern sensory science and chemical profiling, positioning multiple successive infusions as a scientifically grounded model for optimizing both sensory experience and bioactive compound delivery. Future studies integrating diverse tea varieties, brewing parameters, and physiological endpoints will further clarify how traditional preparation methods can be harnessed to maximize health‐relevant benefits.

## Author Contributions

**Xiaoying Zhang**: conceptualization, methodology, data curation, supervision, writing – review and editing, writing – original draft. **Anas Yusuf**: writing – original draft. **Man Xu**: investigation, validation, formal analysis. **Murtala Bindawa Isah**: writing – original draft, validation.

## Funding

This work was supported by the Talent Initiative Project (X20240142) and the Special Project for Scientific Research and Capacity Building (X20240022) at Shaanxi University of Technology, China. Incubation Project on State Key Laboratory of Biological Resources and Ecological Environment of Qinba Areas (SLGPT2019KF04‐04), China.

## Conflicts of Interest

The authors declare no conflicts of interest.

## Supporting information




**Supplementary materials**: jfds71082‐sup‐0001‐SuppMat.docx
