# Supplementary material for: Three Successive Infusions: Scientific Insights Into a Traditional Green Tea Drinking Custom
Source: J Food Sci. 2026 May 6;91:e71082. doi: 10.1111/1750-3841.71082 (PMC13147319; doi:10.1111/1750-3841.71082)
Supplement: Supplementary file 1 — Supplementary materials: jfds71082‐sup‐0001‐SuppMat.docx [file JFDS-91-0-s001.docx]

**Three successive infusions: Scientific insights into a traditional green tea drinking custom**

**Xiaoying Zhang^*1,2,3^, Anas Yusuf^1^, Murtala Bindawa Isah^1,4^, Man Xu^1^,**

^1^ Chinese-German Joint Laboratory for Natural Product Research, Shaanxi International Cooperation Demonstration Base, Hanzhong, 723100 Shaanxi, China

^2^ Centre of Molecular and Environmental Biology (CBMA), Department of Biology, University of Minho, Campus de Gualtar, Braga, 4710-057, Portugal

^3^ Department of Biomedical Sciences, Ontario Veterinary College, University of Guelph, Guelph, ON, Canada

^4^ Department of Biochemistry, Umaru Musa Yar’adua University Katsina, Nigeria

* Correspondence: zhang@bio.uminho.pt

**Supplementary material 1**

**Supplementary Methods**

Determination of mineral and trace elements

To determine the mineral ion content of the sample, 100 mg of the sample was placed in a nitro boiling tube and digested by open-vessel nitric acid digestion with 2 mL of nitric acid solution for 1 hour at 95°C under atmospheric pressure. Ultrapure water was subsequently added, and the volume was adjusted to 20 mL with dd H_2_O. Ion contents were analysed using an inductively coupled plasma emission spectrometer (ICP-OES; PerkinElmer Avio200, MA, USA).

**Supplementary results**

The levels of mineral and trace elements decreased rapidly with successive infusions

Major mineral and trace elements were analysed in all the teas from the infusions, indicating generally rapid decreasing trends from GTI1 to GTI3 for all the elements (Supplementary Table 1). All the trace elements are present in the teas at concentrations within their safe limits as identified by the Chinese National Standard GB 2762–2017 (Xiao et al., 2023).

**Table S1** Mineral and trace element detection in tea sequential green tea infusions (GTI)

| **Element** | **GTI1 (ng/mL)** | **GTI2 (ng/mL)** | **GTI3 (ng/mL)** |
| --- | --- | --- | --- |
| K | 373.23 ± 8.73 | 79.62 ± 3.85 | 34.17 ± 2.58 |
| P | 44.82 ± 1.75 | 8.53 ± 0.59 | 3.71 ± 1.02 |
| Mg | 37.02 ± 1.68 | 5.44 ± 0.26 | 1.69 ± 0.27 |
| Al | 10.36 ± 1.43 | 4.73 ± 3.36 | 4.50 ± 2.44 |
| Mn | 4.16 ± 0.19 | 0.74 ± 0.05 | 0.28 ± 0.03 |
| Na | 0.89 ± 0.09 | 0.18 ± 0.05 | 0.29 ± 0.15 |
| Zn | 0.55 ± 0.04 | 0.16 ± 0.02 | 0.08 ± 0.03 |
| Ni | 0.48 ± 0.01 | 0.20 ± 0.04 | 0.17 ± 0.02 |
| B | 0.09 ± 0.01 | 0.05 ± 0.00 | 0.03 ± 0.00 |
| V | 0.05 ± 0.03 | 0.15 ± 0.06 | 0.17 ± 0.04 |
| Cu | 0.05 ± 0.01 | 0.04 ± 0.02 | 0.05 ± 0.01 |


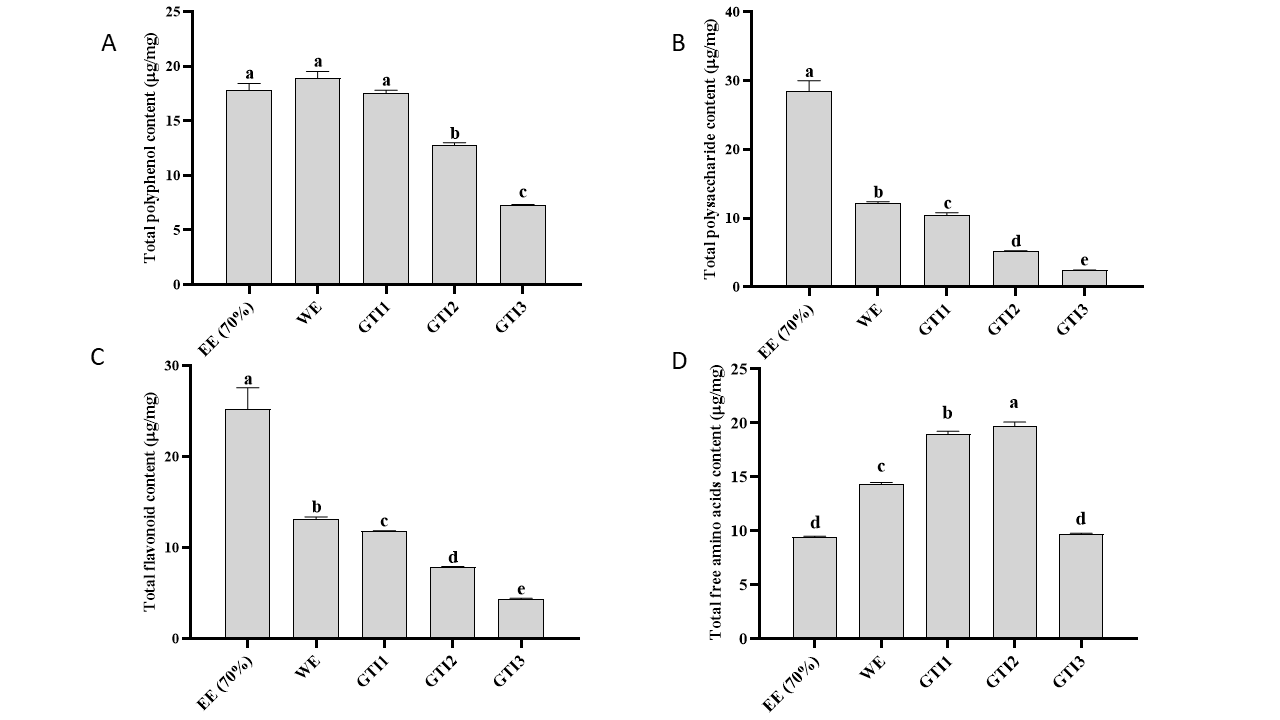


**Figure S1 Quantification of total compounds and amino acids across the tea infusions**

**Notes:** Total polyphenols (A), total polysaccharides (B), total flavonoids (C), and total amino acid (D) across all tea infusions.


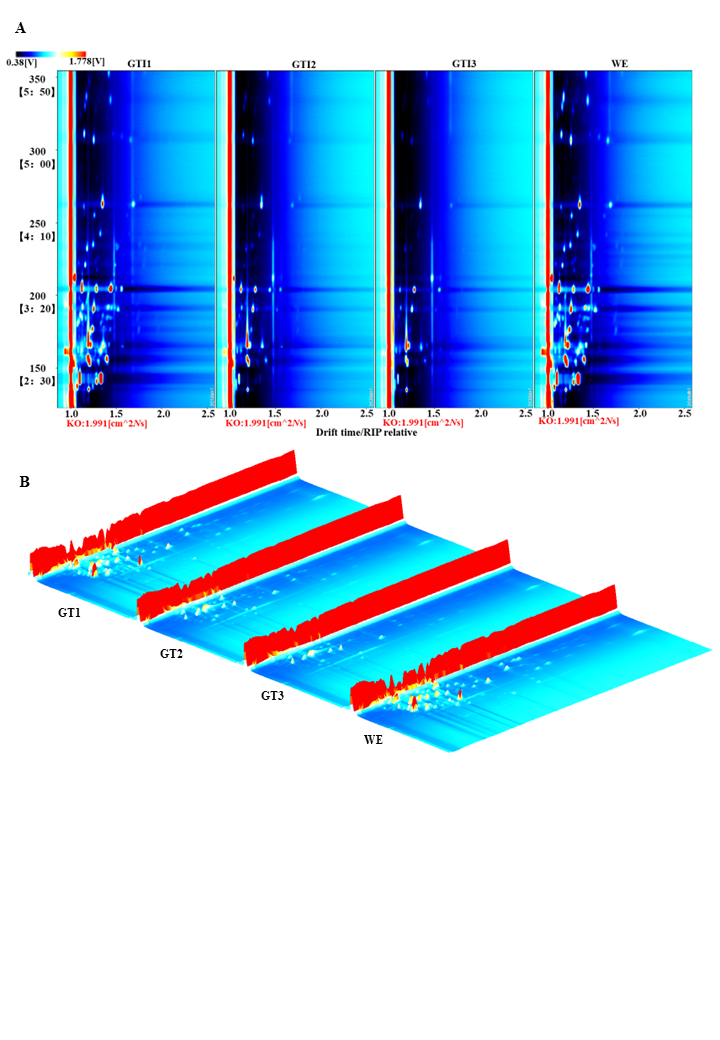


Figure S2 Two-dimensional (2D) spectra of green tea infusions (A) and Three-dimensional (3D) topographic plot of GT1, GT2, and GT3 (B)

**
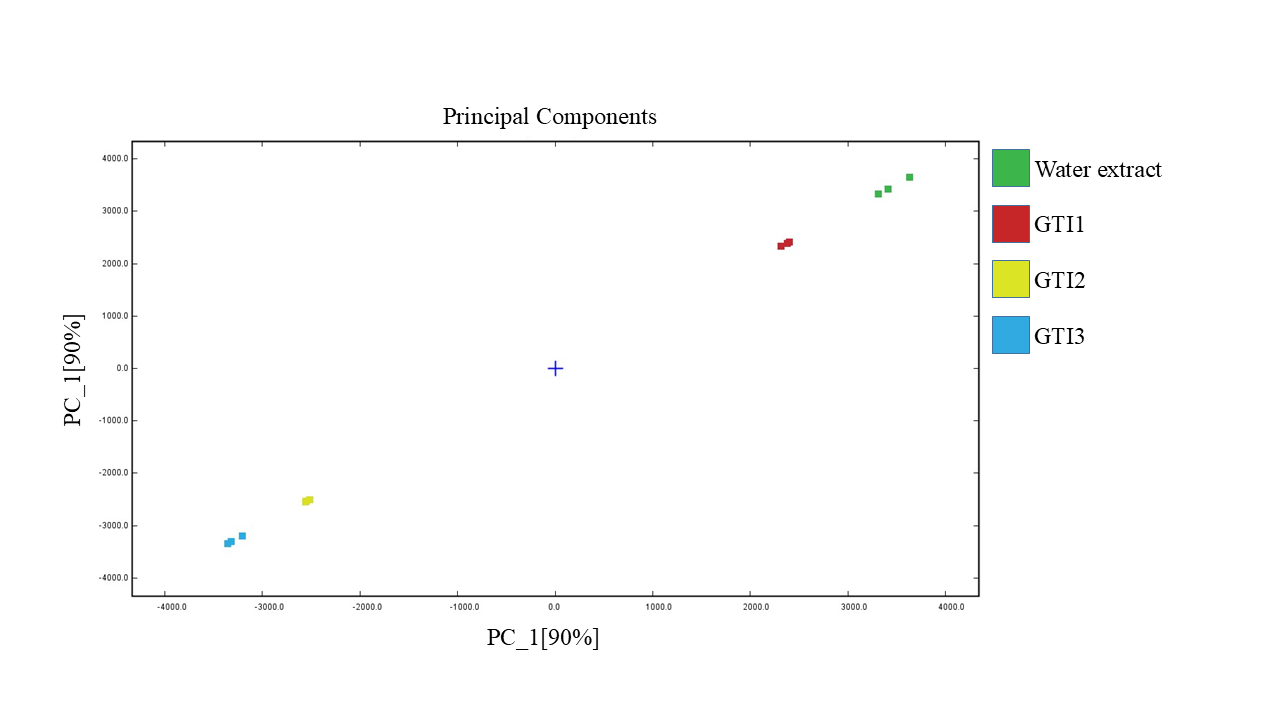
**

**Figure S3 PCA analysis of volatile compounds across the tea infusions**

**
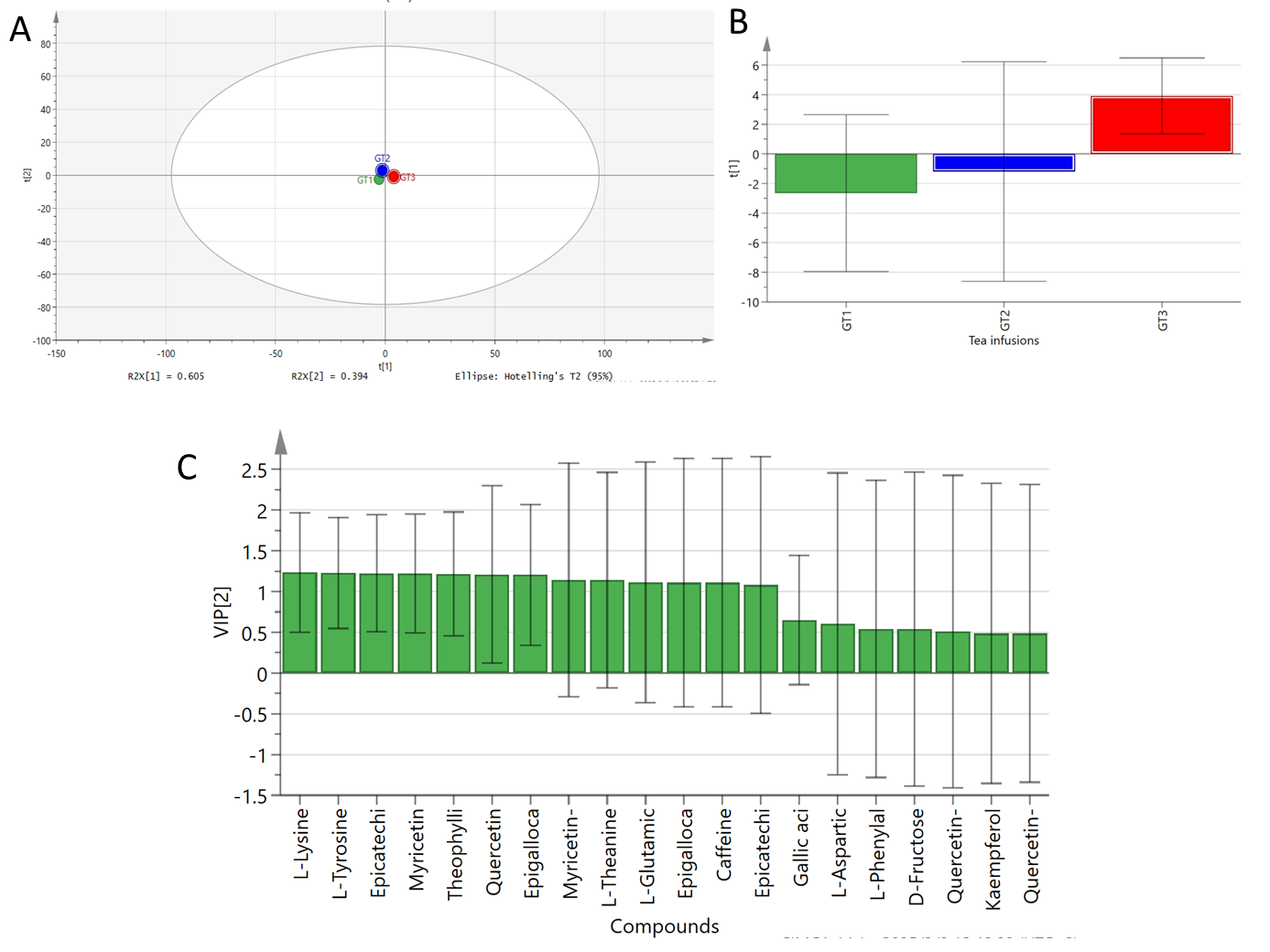
**

**Figure S4 PLSR analysis of tea infusions (GT1–GT3)**

**Notes:** (A) Score plot illustrating sample separation along the first two latent variables; (B) Column plot highlighting progressive sensory differentiation across infusions; (C) VIP scores identifying the compounds most influential in explaining sensory attribute variations.

**Table S2 Volatile compounds and their classifications**

| **Classification** | **Compounds** | **CAS** | **MW** | **Oduor description** |
| --- | --- | --- | --- | --- |
| **Alcohols** | 3-methyl-3-buten-1-ol | 763-32-6 | 86.13 | Fruity and sweetness |
|  | 1-Pentanol | 71-41-0 | 88.15 | Fusel, paint smell |
|  | (3E)-Hexenol | 928-97-2 | 100.16 | Grass fragrance |
|  | 1-Hexanol | 111-27-3 | 102.17 | Light green leaves scent |
|  | 2-Hexanol | 626-93-7 | 102.17 | Wine aroma |
|  | 1-Octen-3-ol | 3391-86-4 | 128.21 | Mushroom, lavender, rose, hay aromas |
|  | (Z)-2-Pentenol | 1576-95-0 | 86.13 | Grass fragrance |
|  | 2-Octanol | 123-96-6 | 130.22 | Spicy |
| **Aldehydes** | (E)-2-Hexenal | 6728-26-3 | 98.14 | Fruit and green leaf aroma |
|  | Pentanal | 110-62-3 | 86.13 | Fermentation odor |
|  | (E)-pent-2-enal | 1576-87-0 | 84.12 | Grass fragrance |
|  | Butanal | 123-72-8 | 72.11 | Spicy flavor |
|  | Heptanal | 111-71-7 | 114.19 | Grass fragrance |
|  | 2-Methylbutanal | 96-17-3 | 86.13 | Fermented aroma |
|  | 3-Methylbutanal (Isovaleraldehyde) | 590-86-3 | 86.13 | Fruity aroma |
| **Ketones** | 2-Butanone | 78-93-3 | 72.11 | Fruity, grassy |
|  | 3-Hydroxy-2-butanone | 513-86-0 | 88.11 | Milk and buttery flavor |
|  | 4-Methyl-3-penten-2-one | 141-79-7 | 98.14 | Honey smell |
|  | 6-Methyl-5-hept-5-en-2-one | 110-93-0 | 126.20 | Fruity and fresh |
|  | 2,3-Pentanedione | 600-14-6 | 100.12 | Sweet butter, caramel, nutty aroma |
|  | 2-Hexanone | 591-78-6 | 100.16 | Fruity aroma |
|  | Cyclopentanone | 120-92-3 | 84.12 | Mint flavor |
|  | Cyclohexanone | 108-94-1 | 98.14 | Mint flavor |
| **Esters** | Methyl acetate | 79-20-9 | 74.08 | Spicy, sweet; aromatic |
|  | Ethyl acetate | 141-78-6 | 88.11 | Fruity aroma |
|  | Ethyl propanoate | 105-37-3 | 102.13 | Fruity aroma |
|  | methyl 3-methylbutanoate | 556-24-1 | 116.16 | Fruity aroma |
| **Carboxylic Acids** | Butanoic acid | 107-92-6 | 88.11 | Pungent odor |
| **Furans** | 2,5-Dimethylfuran | 625-86-5 | 96.13 | Meaty |
|  | furan-2-ylmethyl acetate | 623-17-6 | 140.14 | Fruity aroma |
|  | 2-ethylfuran | 3208-16-0 | 96.13 | Burnt smell |
| **Others** | (methyldisulfanyl)methane | 624-92-0 | 94.2 | Sulfur smell |
|  | Sarin | 107-44-8 | 140.09 |  |
|  | Pyrrolidine tetrahydropyrrole | 123-75-1 | 71.12 | Pungent ammonia odor |
|  | 1-(1H-pyrrol-2-yl)-ethanone | 1072-83-9 | 109.13 | Baked flavor |
|  | 1,2-Dimethoxyethane | 110-71-4 | 90.12 | Ether odor |
|  | methyl furan-2-carboxylate | 611-13-2 | 126.11 | Fruit, mushroom flavor |

Notes: The identification of odour description was based on previous studies (Zhu et al., 2018; Api et al., 2019; Zhu et al., 2021; Zhu et al., 2021; Zhong et al., 2023)

**References**

Xiao, J., Zhang, D., Shen, J., Gong, W., Wu, X., Wang, F., Chen, Y., Li, X., Zheng, Q., Luo, D., & Lu, S. (2023). Trace elements in successive tea infusions made via a brewing method widespread in China: Implications for human exposure. *Journal of Food Composition and Analysis*, *115*, 104989. https://doi.org/10.1016/j.jfca.2022.104989
